# Inhalable Polymeric Nanoparticle Containing Triphenylphosphanium Bromide‐modified Sonosensitizer for Enhanced Therapy of Acute Bacterial Pneumonia

**DOI:** 10.1002/advs.202417469

**Published:** 2025-04-02

**Authors:** Lin Wang, Fangzhou Chen, Nier Wu, Lingfei Hu, Haihua Xiao, Hanchen Zhang, Dongsheng Zhou

**Affiliations:** ^1^ State Key Laboratory of Pathogen and Biosecurity Academy of Military Medical Sciences Beijing 100071 China; ^2^ Northern Medical Branch of PLA General Hospital Beijing 100094 China; ^3^ Beijing National Laboratory for Molecular Sciences Laboratory of Polymer Physics and Chemistry Institute of Chemistry Chinese Academy of Sciences Beijing 100190 China

**Keywords:** bacterial pneumonia, cationic triphenylphosphanium bromide, ESKAPE pathogens, pseudo‐conjugated polymer, sonodynamic therapy

## Abstract

Sonodynamic therapy (SDT) has good feasibility to deeply seated infections, but SDT alone is insufficient being highly effective against multidrug‐resistant (MDR) bacteria. SDT combined with triphenylphosphanium bromide (P^+^Ph_3_Br^−^) is expected to solve this problem. This work develops a pseudo‐conjugated polymer P^FCPS‐P^ containing cationic P^+^Ph_3_Br^−^‐modified sonosensitizer FCPS (FCPS‐P) and ROS‐sensitive thioketal bonds. P^FCPS‐P^ is assembled with DSPE‐mPEG_2000_ to generate nanoparticle NP^FCPS‐P^. FCPS has SDT effect and generates ROS under ultrasound (US) stimulation. ROS triggers the degradation of NP^FCPS‐P^ and release of FCPS‐P, endowing highly favored biosafety. FCPS‐P targets to bacterial surface through electrostatic interaction and achieves bacterial killing under a synergistic action of SDT and P^+^Ph_3_Br^−^. In vitro, NP^FCPS‐P^+US gives >90% inhibition rates against MDR ESKAPE pathogens, moreover, it causes bacterial metabolic disorders including inhibited nucleic acid synthesis, disordered energy metabolism, excessive oxidative stress, and suppressed biofilm formation and virulence. In mice, NP^FCPS‐P^+US exhibits a 99.3% bactericidal rate in *Pseudomonas aeruginosa*‐induced sublethal pneumonia and renders a 90% animal survival rate in lethal pneumonia, and additionally immunological staining and transcriptomics analyses reveal that NP^FCPS‐P^+US induces inhibited inflammatory response and accelerated lung injury repair. Taken together, NP^FCPS‐P^+US is a promising antibiotics‐alternative strategy for treating deeply seated bacterial infections.

## Graphical abstract

Bacterial pneumonia is the sixth leading cause of death. In this study, an inhalable polymeric nanoparticle NP^FCPS‐P^ is developed, which under US stimulation showed high efficacy in the treatment of acute bacterial pneumonia. This nanoplatform would be much less prone to select bacterial resistance and represented a promising measure for antibiotics‐alternative therapy of deeply seated infections.

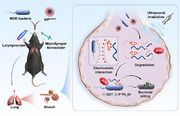

## 1 Introduction

Bacterial pneumonia is the sixth leading cause of death and the only infectious disease in the top ten causes of death.^[^ ^1^ ^]^ ESKAPE pathogens contain the following six pathogenic bacteria *Enterococcus faecium* (*Ec. faecium*), *Staphylococcus aureus* (*S. aureus*), *Klebsiella pneumoniae* (*K. pneumoniae*), *Acinetobacter baumannii* (*A. baumannii*), *Pseudomonas aeruginosa* (*P. aeruginasa*), and *Enterobacteriaceae* species such as *Eb. Hormaechei*,^[^ ^2^ ^]^ and they account for about two‐thirds of the total nosocomial infections worldwide (https://microbenotes.com/eskape‐pathogens‐antimicrobial‐resistance). *S. aureus, P. aeruginasa*, *K. pneumoniae, A. baumannii* represent the most frequent pathogens of hospital‐acquired bacterial pneumonia), ^[^ ^3^ ^]^ which is among the most common nosocomial infections.^[^ ^4^ ^]^ Most ESKAPE isolates are multidrug resistant (MDR) and capable of escaping bactericidal actions of traditional antibiotics, which poses a risk of the ending of Antibiotic Era and urgently prioritizes in need of developing antibiotics‐alternative therapeutic strategies^[^ ^5^ ^]^ such as sonodynamic therapy (SDT).^[^ ^6^ ^]^

As a prerequisite for SDT, sonosensitizer is activated by ultrasound (US) to produce reactive oxygen species (ROS) through cavitation effect and sonoluminescence effect.^[^ ^6^ ^]^ By at least causing protein denaturation and DNA damage, the generated ROS is the main reason for SDT‐based bacterial killing; meanwhile, ultrasonic cavitation endows sonomechanical damage and increased surrounding temperatures acting as additional bactericidal mechanisms.^[^ ^6^ ^]^ SDT generally uses low‐intensity focused US (0.5–3.0 W cm^−2^, 1.0–2.0 MHz), which has minor tissue damage and higher biosafety compared to high‐intensity focused US.^[^ ^7^ ^]^ US takes advantage of superior tissue penetrability, enabling SDT to have great feasibility to deeply seated infections including pneumonia.^[^ ^6^ ^]^

Organic sonosensitizers exhibit good biodegradability and biosafety, but they often suffer from poor water solubility and short blood circulation.^[^ ^9^ ^]^ Liposome or polymer nanocarriers encapsulated with organic sonosensitizers would enhance their controlled release property and pharmacokinetic profile in blood circulation.^[^ ^9^ ^]^ In addition, sonosensitizers are needed to enhance their targeting ability to bacterial cells over mammalian cells.^[^ ^10^ ^]^ Through electrostatic interaction, cationically charged organic sonosensitizers would increase their selectively targeting affinity to bacteria, which are typically more anionic than mammalian cells.^[^ ^10^ ^]^

**Scheme 1:**
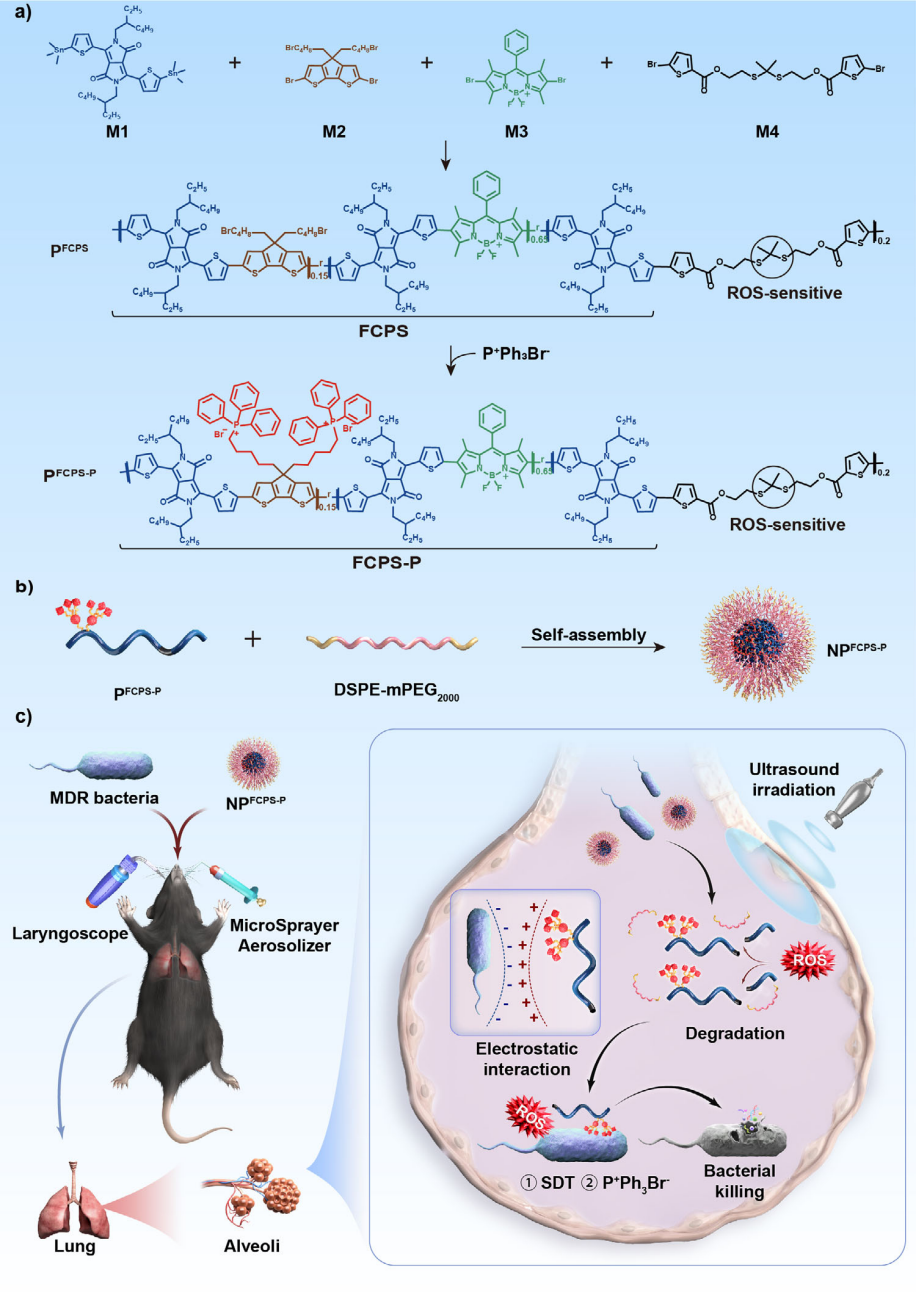
Schematic illustration of synthesis and bactericidal action of NP^FCPS‐P^. a) The polymer P^FCPS^ was synthesized from the four monomers M1, M2, M3, and M4, among which the first three made up the polymer sonosensitizer FCPS while the last one harbored the ROS‐sensitive thioketal bond. Furthermore, P^FCPS‐P^ was obtained by conjugating P^+^Ph_3_Br^−^ to M2 in FCPS. Here, FCPS‐P manifested as P^+^Ph_3_Br^−^‐modified sonosensitizer FCPS. b) P^FCPS‐P^ and DSPE‐mPEG_2000_ were self‐assembled together into the nanoparticle NP^FCPS‐P^. c) NP^FCPS‐P^ was delivered in the lung of mouse via aerosolized intratracheal inoculation. NP^FCPS‐P^ under US stimulation (NP^FCPS‐P^+US) produced high levels of ROS owing to FCPS‐mediated SDT activity. The generated ROS led to the breakage of ROS‐sensitive thioketal bonds in the polymers, resulting in the degradation of NP^FCPS‐P^ and the release of FCPS‐P. This ROS‐responsive degradability endowed the highly favorable biosafety of NP^FCPS‐P^. The electrostatic interaction between cationic FCPS‐P and negatively charged bacteria surfaces would enhance the targeted attack on bacteria. SDT along with P^+^Ph_3_Br^−^ exhibited a synergistic effect for anti‐infection. NP^FCPS‐P^+US represented a highly biocompatible nanoplatform for antibiotics‐alternative treatment of acute bacterial pneumonia.

Inspired by the above principles, we designed a biodegradable pseudo‐conjugated polymer P^FCPS‐P^ composed of ROS‐sensitive thioketal bonds and FCPS‐P, for which a polymer sonosensitizer FCPS was chemically bonded with cationic triphenylphosphanium bromide (P^+^Ph_3_Br^−^) at its side chain (**Scheme** **1**). P^FCPS‐P^ was assembled with commercially available DSPE‐mPEG_2000_ to generate the nanoparticle NP^FCPS‐P^, which could be delivered into the lungs via aerosolized intratracheal inoculation. NP^FCPS‐P^ under US stimulation (NP^FCPS‐P^+US) produced high levels of ROS owing to FCPS‐mediated SDT activity. The action of the generated ROS on ROS‐sensitive thioketal bonds in the polymers resulted in the degradation of NP^FCPS‐P^ and the release of FCPS‐P. This ROS‐responsive biodegradability granted the highly favorable biosafety of NP^FCPS‐P^ at both cellular and animal levels. The cationic charge of FCPS‐P endowed the targeting accumulation of FCPS‐P on the negatively charged cell surface of bacteria, and moreover, the FCPS‐induced SDT worked in synergy with P^+^Ph_3_Br^−^ (known as a classical antimicrobial agent to disrupt bacterial cell membrane^[^ ^11^ ^]^) to afford the enhanced antibacterial efficacy. NP^FCPS‐P^+US gave >90% inhibition rates against ESKAPE pathogens in vitro. NP^FCPS‐P^+US exhibited a 99.3% bactericidal rate in a mouse model of *P. aeruginosa*‐induced sublethal pneumonia and moreover rendered a 90% mouse survival rate in the corresponding lethal pneumonia model. NP^FCPS‐P^+US represented a promising antibiotics‐alternative strategy for the treatment of deeply seated infections caused by MDR bacteria.

## 2 Result and Discussion

### 2.1 Synthesis and Characterizations of NP^FCPS‐P^

There were totally three polymers (P^FCPS^, P^FCPS‐P^, and commercially available DSPE‐mPEG_2000_) and two nanoparticles (NP^FCPS^ and NP^FCPS‐P^) as described in Scheme 1 and Figure S1 (Supporting Information). P^FCPS^ was synthesized from four monomers M1, M2, M3, and M4 via a so‐called Stille reaction (Scheme 1a). M1, M2, and M3 constituted the polymer sonosensitizer FCPS with a molecular weight of 14 KDa. M1 acted to connect M2 and M3. M2 could expand the conjugation system, reduce the electronic cloud density and the orbital energy level, and facilitate the electronic transition of P^FCPS^, thereby enhancing the sonodynamic effect of P^FCPS^. M3, as a classic structural monomer of a sonosensitizer, inherently possesses significant sonodynamic efficacy. M4 contained the ROS‐sensitive thioketal bond, rendering P^FCPS^ biodegradable. P^+^Ph_3_Br^−^ was bonded to the side chain of FCPS to form FCPS‐P with a molecular weight of 15 KDa, and thus FCPS‐P contained cationic triphenylphosphanium bromide known as a classical antimicrobial agent^[^ ^11^ ^]^ and was sill recognized as a polymer sonosensitizer.

The successful synthesis of P^FCPS^ and P^FCPS‐P^ was confirmed by ^1^H NMR (Figures S1, S2, Supporting Information). In the ^1^H NMR spectrum of P^FCPS^, the peaks observed at ≈0.8 and 1.3 ppm can be assigned to the alkane chains of M1 and M2, respectively. The peak near 4.04 ppm corresponds to the methyl group of M3, while the peak ≈1.5 ppm is attributed to the methyl group of M4. Additionally, the peaks in the range of 7.0 to 8.0 ppm are associated with the protons of the benzene ring in M3 and the protons of the thiophene ring in M1. In contrast to P^FCPS^, the ^1^H NMR spectrum of P^FCPS‐P^ shows an additional peak at 7.3 ppm, which is attributed to triphenylphosphine. This result indicated that triphenylphosphine has been successfully grafted onto the side chain of P^FCPS^.

**Figure 1:**
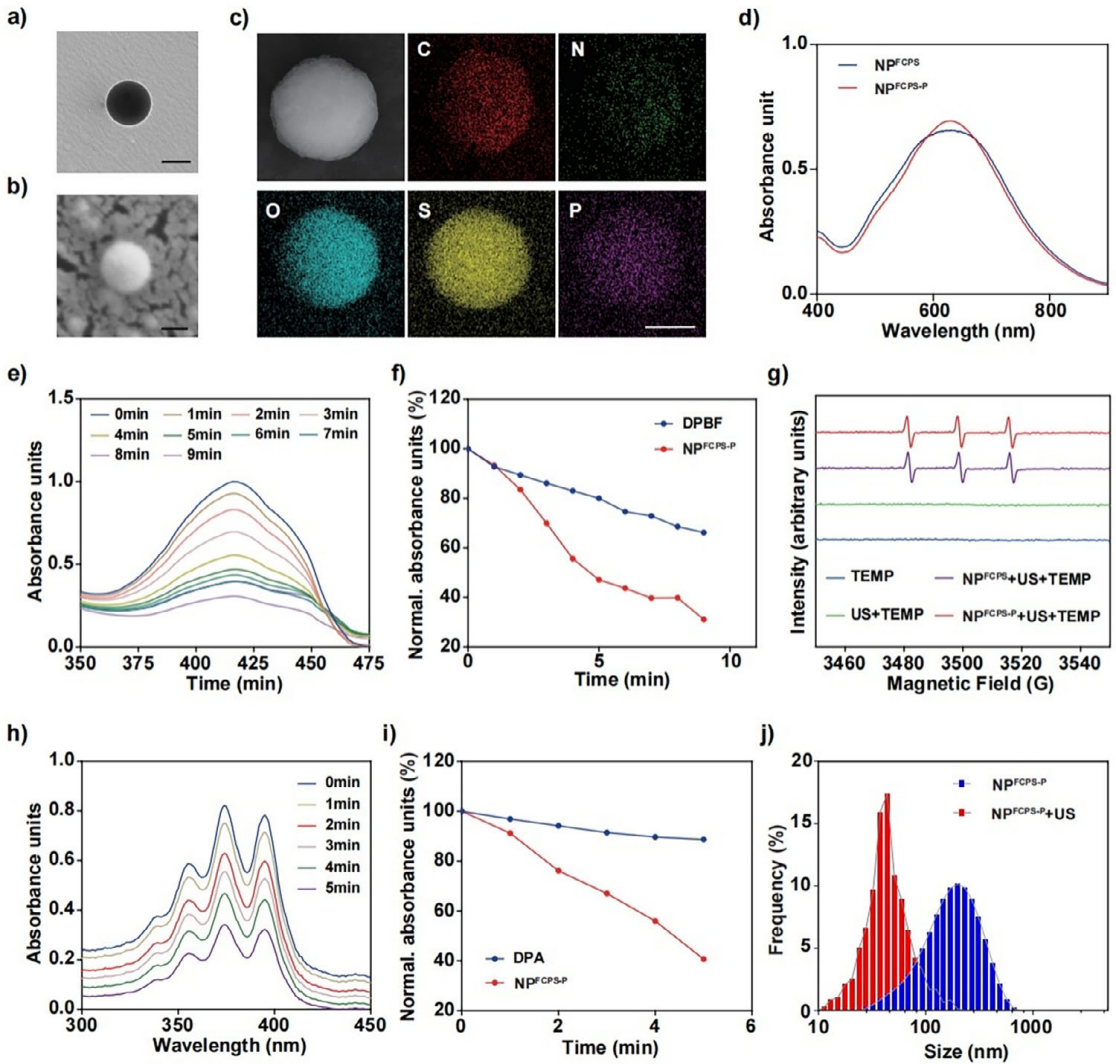
Characterization of NP^FCPS‐P^. a) TEM images of NP^FCPS‐P^. Scale bar = 100 nm. b) SEM images of NP^FCPS‐P^. Scale bar = 100 nm. c) STEM and EDXS images of NP^FCPS‐P^. Scale bar = 50 nm. d) UV–vis‐NIR absorption spectra of NP^FCPS‐P^ and NP^FCPS^. e) Absorption curves of DPBF as a selective trapping agent to detect ROS in the reaction mixture of NP^FCPS‐P^ under US stimulation (1.0 MHz, 1.5 W cm^−2^, 50% duty cycle). f) Trend of UV absorption at 410 nm for the above DPBF‐based detection results. g) ESR spectra demonstrating ^1^O_2_ generation of NP^FCPS‐P^ or NP^FCPS^ with US stimulation (1.5 W cm^−2^, 1.0 MHz, 50% duty cycle, 1 min). h) Absorption curves of DPA as a selective trapping agent to detect ^1^O_2_ in the reaction mixture of NP^FCPS‐P^ under US stimulation (1.0 MHz, 1.5 W cm^−2^, 50% duty cycle). i) Trend of UV absorption at 378 nm for the above DPA‐based detection results. DLS detection of j) size distributions of NP^FCPS‐P^ with or without US stimulation (1.0 MHz, 1.5 W cm^−2^, 50% duty cycle, 6 min).

The hydrophobic polymer P^FCPS‐P^ or P^FCPS^ in the absence of DSPE‐mPEG_2000_ would aggregate in water and could not be assembled into nanoparticles. In order to improve the water solubility of P^FCPS‐P^ or P^FCPS^ and meanwhile to reduce the side effect of P^+^Ph_3_Br^−^, the amphiphilic DSPE‐mPEG_2000_ was used to encapsulate P^FCPS‐P^ or P^FCPS^ to obtain NP^FCPS‐P^ (Scheme 1b) or NP^FCPS^ (Scheme S1, Supporting Information) as the micelle, respectively. P^FCPS‐P^ or P^FCPS^ was located at the core of NP^FCPS‐P^ or NP^FCPS^, respectively. As observed by transmission electron microscopy (TEM) and scanning electron microscopy (SEM), NP^FCPS‐P^ (**Figure** **1**a,b and Figure S3, Supporting Information) or NP^FCPS^ (Figure S4, Supporting Information) presented uniform and complete spherical shape with an average particle size of 118 or 117 nm, respectively. Energy dispersion‐X‐ray spectroscopic scanning (EDXS) confirmed the presence of elements C, N, O, S, and P in NP^FCPS‐P^ (Figure 1c) and P^FCPS^ (Figure S5, Supporting Information). As shown by UV–vis absorption spectrum, NP^FCPS‐P^ and NP^FCPS^ gave almost the same absorption curve with the maximum absorption peak at ≈660 nm (Figure 1d). These demonstrated the successful assembly of NP^FCPS‐P^ and NP^FCPS^.

P^+^Ph_3_Br^−^ alone could not be encapsulated with DSPE‐mPEG_2000_ into a nanoparticle. Each of M1, M2 and M3 could be packaged with DSPE‐mPEG_2000_ to obtain NP^M1^, NP^M2^, and NP^M3^, respectively, and their SDT‐mediated ROS production ability was determined as below; as expected, the absorbance of NP^M3^ was decreased by 24%, which was higher than the 11%, 14%, and 11% decrease values of NP^M1^, NP^M2^, 1,3‐diphenylisobenzofuran (DPBF) blank control, respectively (Figure S6, Supporting Information). These results confirmed M3 as a core determinant of sonodynamic effect.

To further validate the sonodynamic effect of NP^FCPS‐P^ or NP^FCPS^, 2 mm DPBF as the ROS probe was mixed with 20 µg mL^−1^ NP^FCPS‐P^ or NP^FCPS^ followed by US stimulation for 0 to 9 min. As determined by a multimode reader, the absorption peak of NP^FCPS‐P^ at 410 nm was decreased rapidly under US and the peak intensities was gradually decreased as the prolonging of US stimulation times (Figure 1e); after 9 min of US stimulation, the absorbance of NP^FCPS‐P^ decreased by 69%, which was higher than the 34% decrease of DPBF blank control (Figure 1f). NP^FCPS^ showed similar results (Figure S7, Supporting Information). These proved that NP^FCPS‐P^ and NP^FCPS^ had almost the same SDT‐mediated ROS production ability.

The ability of NP^FCPS‐P^ or NP^FCPS^ to produce ^1^O_2_ and •OH under US stimulation was systematically characterized by a series of electron spin resonance (ESR) experiments. First, 3 µg mL^−1^ 2,2,6,6‐tetramethylpiperidine (TEMP) and 10 µg mL^−1^ 5,5‐dimethyl‐1‐pyrroline‐N‐oxide (DMPO), as the capture agents for ^1^O_2_ and •OH, respectively, were mixed with 100 µg mL^−1^ of NP^FCPS‐P^ or NP^FCPS^ followed by US stimulation for 1 min. NP^FCPS‐P^+US produced a strong ^1^O_2_ signal peak (Figure 1g), while the signal peak of •OH was not detected (Figure S8, Supporting Information). NP^FCPS^+US showed similar results (Figure 1g and Figure S8, Supporting Information). These disclosed that NP^FCPS‐P^ and NP^FCPS^ produced ^1^O_2_ but not •OH upon US stimulation. Second, 1 mg mL^−1^ 9,10‐dibenzoanthraquinone (DPA) as the ^1^O_2_‐specific probe was mixed with 20 µg mL^−1^ NP^FCPS‐P^ or NP^FCPS^, and the peak intensity at 378 nm was detected after US stimulation for 0 to 5 min to verify the effect of ^1^O_2_ production. The absorption peak of NP^FCPS‐P^ (Figure 1h) was decreased rapidly under US and the peak intensities at 378 nm were gradually decreased with the prolonging of US stimulation times; after 5 min of US stimulation, the absorbance of NP^FCPS‐P^ decreased by 59%, which was higher than the 11% decrease of DPA blank control (Figure 1i). NP^FCPS^+US showed similar results (Figure S9, Supporting Information). These demonstrated that the production of ^1^O_2_ by NP^FCPS‐P^+US and NP^FCPS^+US was time‐dependent. Third, 10 µg mL^−1^ methylene blue (MB) as the •OH‐specific capture agent was mixed with 20 µg mL^−1^ NP^FCPS‐P^ or NP^FCPS^, and the effect of •OH production was characterized by detecting its peak intensity at 664 nm after US stimulation for 0 to 5 min. For both NP^FCPS‐P^ and NP^FCPS^, the peak intensity at 664 nm showed no obvious change with the prolonging of US stimulation times (Figure S10, Supporting Information), confirming that there was no •OH production as detected by ESR above. Collectively, NP^FCPS‐P^ and NP^FCPS^ under US stimulation could produce ^1^O_2_ in a time‐dependent manner but they did not generate •OH. This is attributed to the ability of the sonosensitizer to convert O_2_ to ROS after absorbing the energy of ultrasound.

The sensitivity of NP^FCPS‐P^ or NP^FCPS^ to ROS was examined in vitro. First, as shown by gel permeation chromatography (GPC) curves, the molecular weight of P^FCPS‐P^ (3 mg mL^−1^) was gradually decreased from 14.7 into 1.5 KDa after co‐incubation with 10 mm H_2_O_2_ from 0 to 8 h, indicating that the ROS‐sensitive group of P^FCPS‐P^ was broken under the action of H_2_O_2_ (Figure S11a, Supporting Information). P^FCPS‐P^ showed similar results (Figure S11b, Supporting Information). Second, as observed by TEM and SEM, NP^FCPS‐P^ (Figure S12a,b, Supporting Information) and NP^FCPS^ (Figure S12c,d, Supporting Information) after US stimulation for 6 min became smaller in size but still had uniform spherical shapes. Third, as measured by dynamic light scattering (DLS), the average diameter of NP^FCPS‐P^ after US stimulation for 6 min was changed from 115.9 to 53.65 nm (Figure 1j), while its polydispersity index (PDI) had no obvious change (Figure S13a, Supporting Information), and its average Zeta potential was changed from ‐24.1 to ‐33.2 mV (Figure S13b, Supporting Information). NP^FCPS^ gave similar DLS results (Figure S14, Supporting Information). Collectively, US treatment of NP^FCPS‐P^ or NP^FCPS^ would lead to the degradation of NP^FCPS‐P^ or NP^FCPS^, which would include the breakage of thioketal bonds, the release of FCPS‐P or FCPS, respectively, and the dispersing of DSPE‐mPEG_2000_ in the system (Figures S13, S14, Supporting Information). However, after the termination of US stimulation, the dispersed DSPE‐mPEG_2000_ would re‐encapsulate the released FCPS‐P or FCPS into new nanoparticles with smaller sizes and lower negative charges (Figures S13, S14, Supporting Information). To verify this re‐encapsulation, P^FCPS‐P^ or P^FCPS^ was pre‐treated by 10 mm H_2_O_2_ for 24 h to respectively obtain the released FCPS‐P or FCPS in the system; subsequently, DSPE‐mPEG_2000_ was used to repackage each H_2_O_2_‐treated system, forming NP^FCPS‐P+H2O2^ or NP^FCPS+H2O2^, respectively; as expected, the characterization results (Figure S15, Supporting Information) showed that the particle size and potential of this repackaged NP^FCPS‐P+H2O2^ or NP^FCPS+H2O2^ were comparable to the above US‐treated NP^FCPS‐P^ or NP^FCPS^, respectively. Taken together, NP^FCPS‐P^ possessed excellent sonodynamic activity to generate abundant ^1^O_2_ upon US stimulation, and the generated ROS promoted the degradation of NP^FCPS‐P^ and the release of FCPS‐P.

### 2.2 Antibacterial Efficacy of NP^FCPS‐P^ Against MDR ESKAPE Pathogens in vitro

**Figure 2:**
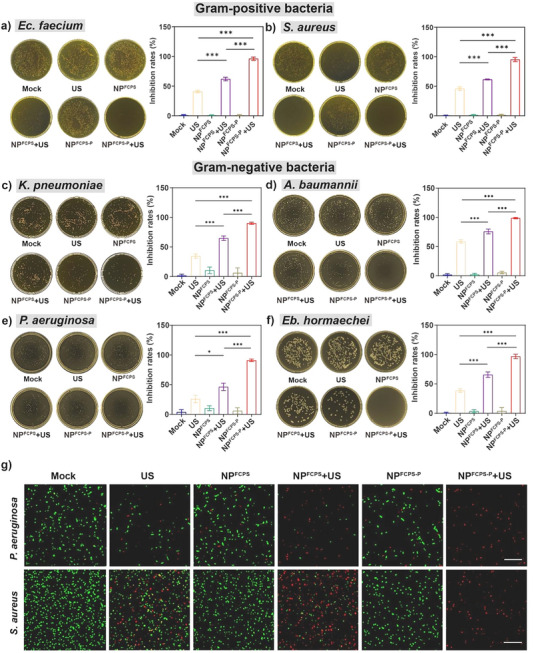
Antibacterial efficacy against ESKAPE pathogens in vitro. US stimulation: 2.0 W cm^−2^, 1.0 MHz, 50% duty cycle, 15 min for Gram‐positive bacteria; 1.5 W cm^−2^, 1.0 MHz, 50% duty cycle, 6 min for Gram‐negative bacteria. a–f) Representative photographs of plate count agars for ESKAPE pathogens post‐treatment, and corresponding statistical analysis of bacterial counts. Data are presented as mean ± standard deviation (SD) (n = 3). ns (not significant): *P* ≥ 0.05, ^*^: *P* < 0.05, ^**^: *P* < 0.01, ^***^: *P* < 0.001, one‐way analysis of variance (ANOVA) with Tukey's multiple comparisons test. g) Representative images (n = 3) of live/dead bacteria post‐treatment as viewed by confocal laser scanning microscopy (CLSM). Red and green fluorescence signals correspond to dead and live bacterial cells, respectively. Scale bar = 20 µm.

The clinical MDR ESKAPE isolates, including *Ec. faecium* HJP554,^[^ ^12^ ^]^ *S. aureus* USA300‐FPR3757,^[^ ^12^ ^]^ *K. pneumoniae* ATCC BAA‐2146,^[^ ^13^ ^]^ *A. baumannii* LAC‐4,^[^ ^14^ ^]^ *P. aeruginosa* F291007,^[^ ^12^ ^]^ and *Eb. hormaechei* ATCC BAA‐2082 (https://www.atcc.org/products/baa‐2082) were subjected to antibacterial experiments in vitro by setting a total of 6 treatment groups namely Mock, US, NP^FCPS^, NP^FCPS^+US, NP^FCPS‐P^, and NP^FCPS‐P^+US (**Figure** **2**). As determined by plate counting method (Figure 2a to 2f), the three control groups Mock, NP^FCPS^, and NP^FCPS‐P^ had no obvious inhibitory activity against all ESKAPE bacteria, denoting that the nanoparticles alone had no bactericidal effect. The NP^FCPS^+US group (SDT alone) showed higher bactericidal activity than the US group. Compared to all the other groups, the NP^FCPS‐P^+US group (SDT plus P^+^Ph_3_Br^−^) gave the highest inhibition rates (96.07%, 91.79%, 90.05%, 98.76%, 90.93% and 96.85%, respectively) against *E. faecium, S. aureus, K. pneumoniae, A. baumannii, P. aeruginosa* and *Eb. hormaechei*. It should be noted that higher irradiances (W cm^−2^) and longer stimulation times were needed to achieve >90% inhibition rates against Gram‐positive bacteria compared to Gram‐negative ones, most likely because Gram‐positive bacteria had thicker cell walls and thus were more tolerant to US. *P. aeruginosa* and *S. aureus* were chosen as the representatives of Gram‐negative and Gram‐positive ESKAPE bacteria, respectively, to explore the synergistic antibacterial effect of SDT and P^+^Ph_3_Br^−^. Based on the inhibition rates, the synergy factor Q values (calculation formula and evaluation criteria shown in Supporting Information) were calculated at 1.34 and 1.16 for these two bacteria, respectively. These two calculated Q values were >1.15, a cutoff indicator of “strong synergy”.^[^ ^15^ ^]^ Furthermore, live/dead dual fluorescent dye staining, for which the green dye SYTO‐9 and the red dye propidium iodide (PI) differentially stained live and dead bacteria, respectively, were performed for *P. aeruginosa* and *S. aureus* post‐treatment (Figure 2g). A large number of dead bacterial cells and a small number of survived cells were observed in the NP^FCPS^+US group, while almost all bacteria died in the NP^FCPS‐P^+US group, reconfirming the synergy. In conclusion, under the synergistic action of SDT and P^+^Ph_3_Br^−^, NP^FCPS‐P^+US in vitro gave the best and highly effective bactericidal action against MDR ESKAPE pathogens especially Gram‐negative bacteria.

### 2.3 Mechanisms of Bactericidal Action of NP^FCPS‐P^ in vitro

**Figure 3:**
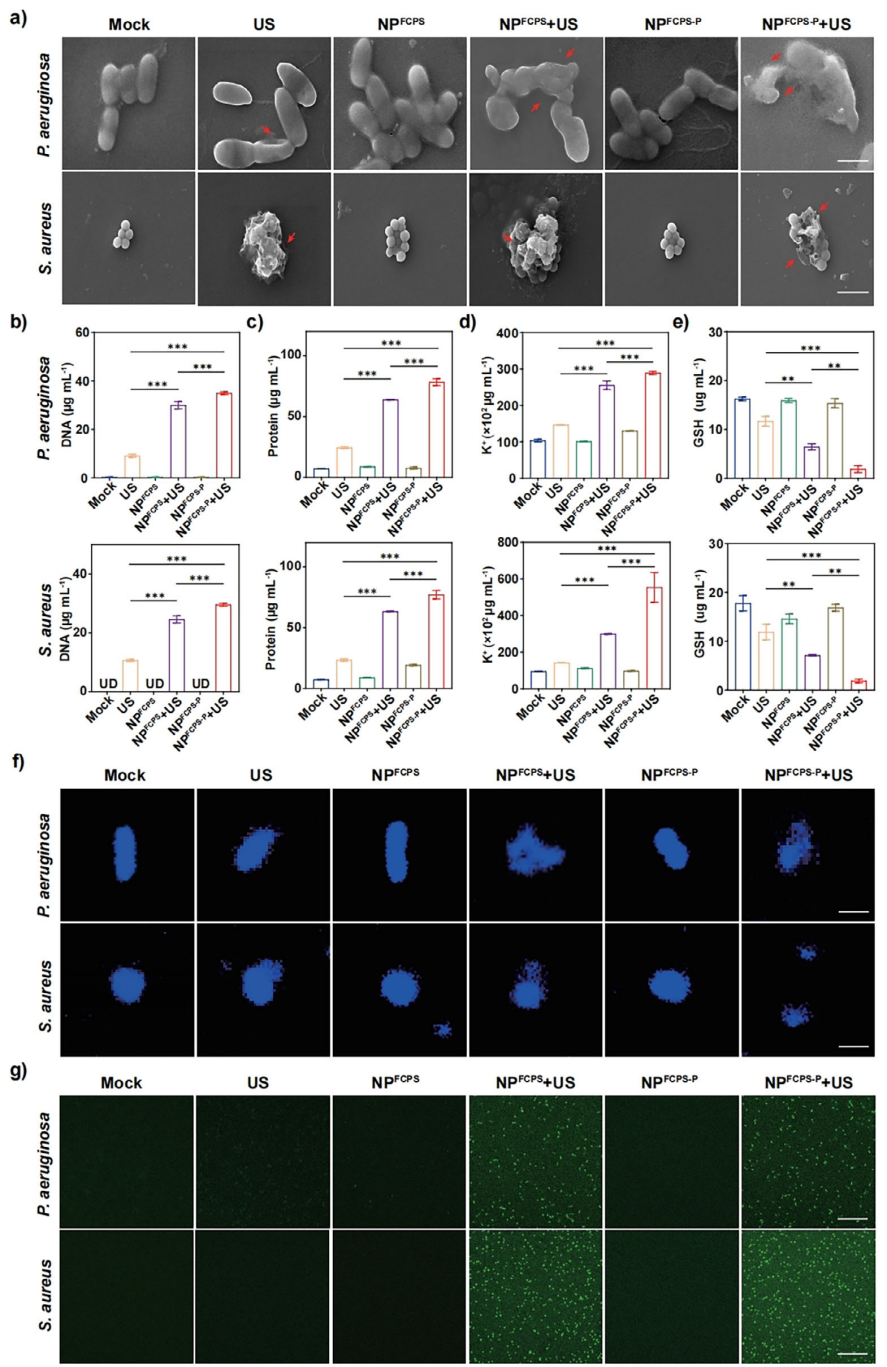
Bacterial morphological and biochemical view of antibacterial mechanisms in vitro. US stimulation: 2.0 W cm^−2^, 1.0 MHz, 50% duty cycle, 15 min for Gram‐positive bacteria; 1.5 W cm^−2^, 1.0 MHz, 50% duty cycle, 6 min for Gram‐negative bacteria. a) SEM images of *P. aeruginosa* and *S. aureus* post‐treatment. Red arrows represent typical disruptions of bacterial morphology. Scale bar = 1 µm. b) DNA, c) Protein, d) K^+^ and e) GSH contents in supernatants of *P. aeruginosa* and *S. aureus* cultures post‐treatment. f) CLSM images of DAPI‐stained bacterial DNAs post‐treatment. Scale bar = 1 µm. g) Fluorescence images of *P. aeruginosa* and *S. aureus* post‐treatment indicating the generation of ROS. The scale bar is 100 µm. Data are presented as mean ± SD (n = 3). ns: *P* ≥ 0.05, ^*^: *P* < 0.05, ^**^: *P* < 0.01, ^***^: *P* < 0.001, one‐way ANOVA with Tukey's multiple comparisons test.

Cell membrane damages and destructive biochemical changes of *P. aeruginosa* and *S. aureus* post‐treatment with the above 6 groups were explored (**Figure** **3**). First, bacterial morphological shrinkage and concavity were observed by SEM (Figure 3a) and TEM (Figure S16, Supporting Information). Second, leakage of bacterial cytoplasmic contents, which were mainly attributed to cell membrane damages,^[^ ^15^ ^]^ was examined by detecting DNA, protein, and K^+^ contents in the supernatants of bacterial cultures (Figure 3b‐d). GSH levels in bacterial cultures were detected by the Reduced GSH Content Assay Kit (Figure 3e), for which the detected decrease in GSH corresponded to weaken ROS defense along with increased oxidative stress in bacterial cells.^[^ ^15^ ^]^ Third, DNAs of bacterial cell nucleus were stained with the blue fluorescence dye 4′,6‐diamidino‐2‐phenylindole (DAPI) (Figure 3f), which was used as the negative indicator of bacterial DNA damage.^[^ ^15^ ^]^ Fourth, ROS levels were detected by using the ROS‐specific green fluorescent probe 2′,7′‐dichlorofluorescein diacetate (DCFH‐DA) (Figure 3g and Figure S17, Supporting Information), which was used as the positive indicator of SDT‐based ROS generation levels in bacterial cultures.^[^ ^15^ ^]^ As shown by the above 4 different assays, a total of 5 detection indexes (namely bacterial morphological shrinkage/concavity, leakage of bacterial cytoplasmic contents, bacterial DNA damage, elevated ROS level in bacterial culture, and decreased GSH level in bacterial culture) were positively detected for the 3 treatment groups NP^FCPS‐P^+US, NP^FCPS^+US, and US, and moreover all these 5 indexes gave the same statistically different trend: NP^FCPS‐P^+US > NP^FCPS^ +US > US; by contrast, no obvious positive results were detected for all the other treatment groups.

**Figure 4:**
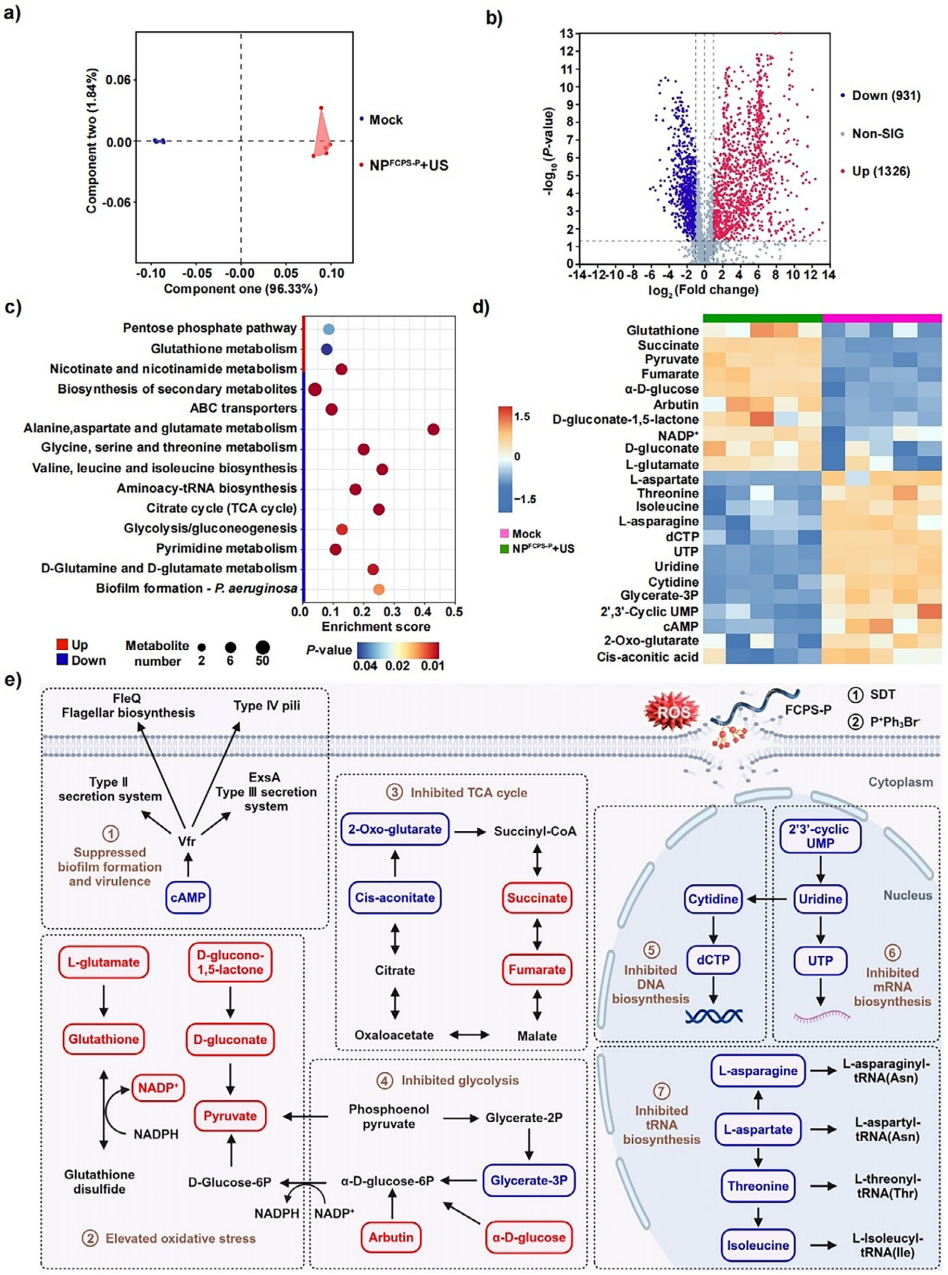
Metabolomics analysis of *P. aeruginosa* after treatment. a) PCA plot of clustered datasets between the two treatment groups NP^FCPS‐P^+US and Mock. US represents US stimulation (1.5 W cm^−2^, 1.0 MHz, 50% duty cycle, 8 min). b) Volcano plot of DRMs identified for NP^FCPS‐P^+US compared to Mock; Up: Up‐regulated metabolites. Down: Down‐regulated metabolites. c) KEGG pathway enrichment analysis of DRMs. d) Heat maps of selected DRMs, and e) diagrams of related dysregulated metabolic pathways. Red represents up‐regulation and blue stands for down‐regulation. Experiments were performed with 5 independent biological replicates.

The metabolomes of *P. aeruginosa* were compared between the NP^FCPS‐P^+US group and the Mock group (**Figure** **4**). First, as disclosed by principal component analysis (PCA), the samples in each treatment group were densely distributed, indicating the existence of limited variation within each group; by contrast, the two groups of data fell into two clusters far apart from each, denoting a high level of difference between the two groups (Figure 4a). Second, as shown in the Volcano plot (Figure 4b) and histogram (Figure S18, Supporting Information), a total of 2257 differential metabolites (DRMs) were identified for NP^FCPS‐P^+US relative to Mock, of which 931 were down‐regulated and 1326 were up‐regulated. Third, KEGG (Kyoto Encyclopedia of Genes and Genomes) analysis (Figure 4c) enriched 11 down‐regulated and 3 up‐regulated pathways, overall illustrating how NP^FCPS‐P^+US induced the obstruction of bacterial virulence, growth and proliferation. Fourth, 10 up‐regulated and 13 down‐regulated metabolites were arbitrarily selected as the representative DRMs for clustering into a heat map (Figure 4d), and moreover, these 23 selected DRMs could be assigned into a dysregulated metabolic pathway map (Figure 4e). cAMP was down‐regulated, which would affect the activity of Vfr as a master positive regulator of biofilm formation and virulence factors in *P. aeruginosa*.^[^ ^16^ ^]^ Up‐regulation of L‐glutamate, glutathione, and NADP^+^ indicated the enhancement of GSH biosynthesis, which would be a compensatory increase of GSH for resistance to SDT‐generated ROS.^[^ ^17^ ^]^ Up‐regulation of D‐gluconate‐1,5‐lactone, D‐gluconate, and pyruvate denoted the elevation of the pentose phosphate pathway (PPP), which would promote bacterial tolerance to oxidative stress by providing NADPH.^[^ ^18^ ^]^ Down‐regulation of glycerate‐3P would act as a metabolic switch to inhibit central carbon pathways such as glycolysis and the TCA cycle, redirecting the carbohydrate flux to PPP to produce more NADPH and GSH to counteract oxidative stress.^[^ ^19^ ^]^ In addition, up‐regulation of succinate in the TCA cycle was generally recognized as a biomarker of oxidative stress.^[^ ^20^ ^]^ Down‐regulation of pyrimidine metabolism‐related 2′,3′‐cyclic UMP, uridine, cytidine, dCTP, and UTP would interfere with DNA and mRNA biosynthesis,^[^ ^21^ ^]^ while down‐regulation of L‐asparagine, L‐aspartate, threonine, and isoleucine would affect multiple tRNA biosynthesis pathways.^[^ ^22^ ^]^ Sixth, through ROC (Receiver Operating Characteristic) curve analysis (Figure S19, Supporting Information), an AUC (area under ROC curve) value of 0.96 was detected for putrescine. This was larger than the cutoff value 0.9.^[^ ^23^ ^]^ The up‐regulated putrescine could be used as a biomarker for predicting the pathological state of *P. aeruginosa* treated with SDT plus P^+^Ph_3_Br^−^, which was consistent with our previous observation for *P. aeruginosa* treated with PTT (photothermal therapy) plus CDT(chemodynamic therapy).^[^ ^24^ ^]^ In conclusion, NP^FCPS‐P^+US leads to suppressed biofilm formation and virulence, excessive ROS production and enhanced oxidative stress, inhibited glycolysis and TCA cycle and inhibited DNA, mRNA, and tRNA biosynthesis in bacteria.

### 2.4 Biosafety and Therapeutic Feasibility of NP^FCPS‐P^

The biological targeting, biosafety, and therapeutic feasibility of NP^FCPS‐P^ were validated prior to in vivo anti‐infective therapy experiments. First, to evaluate the targeting affinity of NP^FCPS‐P^ to bacteria in vitro (Figure S20, Supporting Information), the mixture of *P. aeruginosa* and BEAS‐2B cells was treated by 20 µg mL^−1^ NP^FCPS‐P^ or NP^FCPS^ with or without US stimulation (1.5 W cm^−2^, 1.0 MHz, 50% duty cycle, 2 min). In NP^FCPS‐P^+US group, FCPS‐P was significantly aggregated around *P. aeruginosa* rather than BEAS‐2B cells. After US‐triggered degradation, NP^FCPS‐P^ released positively charged FCPS‐P that would accumulate on the bacterial cell surface through positive and negative electric absorption, achieving the selectively targeting affinity to bacteria. Second, the cytotoxicity of NP^FCPS‐P^ on mouse embryonic normal fibroblast cell line NIH‐3T3 and human ovarian normal epithelial cell line IOSE‐80 was detected by Cell Counting Kit‐8 (CCK‐8) assay (Figure S21, Supporting Information); when cells were incubated with 100 µg mL^−1^ NP^FCPS‐P^ for 24 h (none of US stimulation was added), the cell viability of each cell line reached >80%.^[^ ^12^, ^15^, ^25^ ^]^ In addition, the cytotoxicity of NP^FCPS‐P^ on NIH‐3T3 or IOSE‐80 cells in the presence of 100 µg mL^−1^ NP^FCPS‐P^ together with US stimulation (1.5 W cm^−2^, 1.0 MHz, 50% duty cycle, 6 min) was also tested (Figure S22, Supporting Information), and the results showed that the cell viability of each cell line still reached >80%. Third, through hemolysis test, mouse red blood cells (RBCs) after incubation with 100 µg mL^−1^ NP^FCPS‐P^ for 4 h (none of US stimulation was added) gave the hemolysis rate was <5%^[^ ^12^, ^15^, ^25^ ^]^ (Figure S23, Supporting Information). In addition, the hemolysis rate of RBCs in the presence of 100 µg mL^−1^ NP^FCPS‐P^ together with US stimulation (1.5 W cm^−2^, 1.0 MHz, 50% duty cycle, 6 min) was also measured (Figure S24, Supporting Information), and the results showed that the hemolysis rate was still <5%. Fourth, IVIS spectral in vivo imaging system (Figure S25, Supporting Information) was used to observe the fluorescence distributions in infection‐free mice, which were treated with the 6 groups Mock, US, NP^FCPS^, NP^FCPS^+US, NP^FCPS‐P^, and NP^FCPS‐P^+US (for which NP^FCPS^ or NP^FCPS‐P^ was encapsulated with the red fluorescent dye Cy7.5) using the same route and dose as anti‐infective therapy experiments. On day 0 post‐treatment, much more uniform fluorescence distribution in the lungs was observed for NP^FCPS^+US (compared to NP^FCPS^) or NP^FCPS‐P^+US (relative to NP^FCPS‐P^), indicating that nanoparticles could be accurately delivered into the lungs and US‐promoted the dispersion of nanoparticle in the lungs. The fluorescence was decreased as time went on, indicating that the nanoparticle was gradually metabolized in the body. Fifth, infection‐free mice were treated with the above 6 groups, each of which was co‐delivered with DAPI as a positive control and DCFH‐DA for the detection of ROS (Figure S26, Supporting Information). Compared to the other groups, the NP^FCPS^+US and NP^FCPS‐P^+US groups showed no obvious difference in DCFH‐DA green fluorescence but gave much stronger green fluorescence compared to the other groups, indicating that ROS could be efficiently produced by NP^FCPS^ or NP^FCPS‐P^ upon the US in the lungs. Sixth, infection‐free mice were treated with the above 6 groups, along with the lipopolysaccharide (1 mg kg^−1^) group as the positive control; hematoxylin and eosin (H&E) staining of the tissues from heart, liver, spleen, lung, and kidney on day 6 post‐treatment showed that there was no inflammation and pathological lesions (Figure S27, Supporting Information). For the lipopolysaccharide group, significant inflammatory cell infiltration was observed in lung tissues but not in any of the other four organs. Seventh, as determined by blood biochemical tests on day 6 post‐treatment, the two primary liver function indexes alanine aminotransferase (ALT) and aspartate aminotransferase (AST), and the two primary renal function indexes blood urea nitrogen (BUN) and creatinine (CREA) showed no abnormal changes (Figure S28, Supporting Information). In summary, NP^FCPS‐P^ showed the selectively targeting affinity to bacteria in vitro, and the highly favored biosafety at both cellular and animal levels; US promoted the diffusion of delivered NP^FCPS‐P^ and its CDT‐based ROS generation ability in the lungs.

### 2.5 Therapeutic Efficacy Against Acute Bacterial Pneumonia in Mice

**Figure 5:**
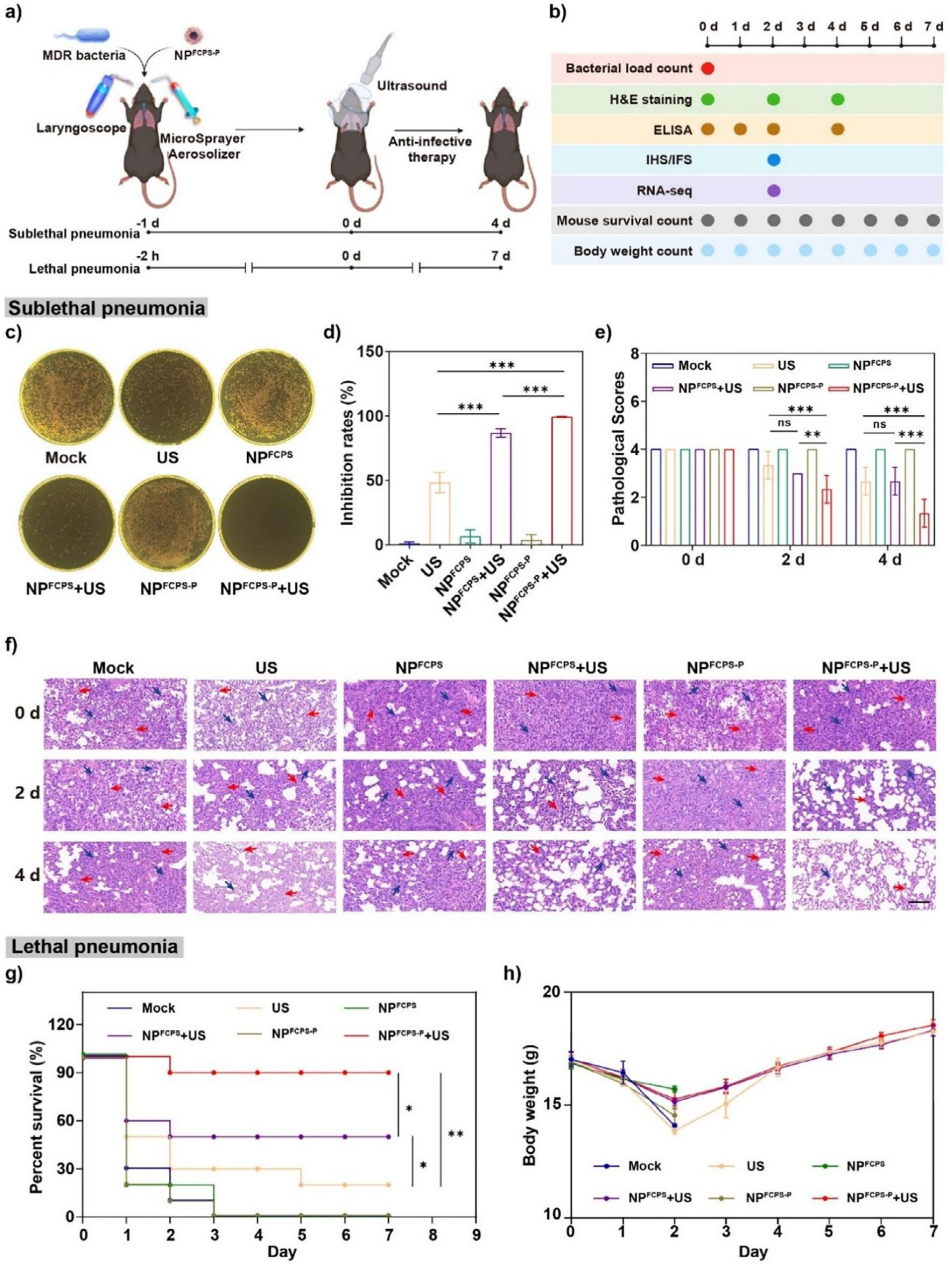
Therapeutic efficacy against *P. aeruginosa*‐induced lung infections in mice. US stimulation: 1.0 MHz, 1.5 W cm^−2^, 50% duty cycle, 2 min. a) Schematic illustration of pulmonary delivery, infection establishment, and anti‐infective therapy. b) Timing diagram for various tests post‐therapy. c) Representative photographs of plate count agars for the lungs once infective therapy was applied, and d) corresponding statistical analysis of bacterial load counts. Data are presented as mean ± SD (n = 6). e) Pathological scores of lung tissues (n = 3) on days 0, 2, and 4 post‐therapy. ns: *P* ≥ 0.05, ^*^: *P* < 0.05, ^**^: *P* < 0.01, ^***^: *P* < 0.001, one‐way ANOVA with Tukey's multiple comparisons test. f) Representative photographs of H&E staining of the lungs. Scale bar = 100 µm. Blue arrows denote inflammatory cells such as neutrophils, macrophages, and lymphocytes. Red arrows stand for local bleeding. g) Survival curves and h) body weights of infected mice post‐therapy. Data are presented as mean ± SD (n = 10). ns: *P* ≥ 0.05, ^*^: *P* < 0.05, ^**^: *P* < 0.01, ^***^: *P* < 0.001, log‐rank Mantel–Cox test.

*P. aeruginosa* F291007, belonging to the high‐risk *P. aeruginosa* ST235 clone characteristic of MDR and hypervirulence,^[^ ^26^ ^]^ was used as the representative to infect mice. Therapeutic efficacy against acute lung infections in mice was evaluated by the programmed procedures including i) co‐delivery of *P. aeruginosa* and nanoparticle via aerosolized intratracheal inoculation, ii) establishment of bacterial infection, and iii) applying of US stimulation to lunch one‐time anti‐infective therapy with the above 6 groups (**Figure** **5**a), which was followed by iv) sampling at different days post‐therapy for subsequent microbiological, immunological, and transcriptomic experiments (Figure 5b). This co‐delivery strategy was employed in our previous study,^[^ ^1^ ^]^ and its reasonability was based on the following three aspects. First, the in vitro bactericidal experiment (Figure 2) proved that NP^FCPS‐P^ or NP^FCPS^ in the absence of US stimulation has no killing effect on bacteria. US stimulation was applied at 24 h post co‐delivery to lunch anti‐infective therapy (Figure 5a), and therefore this co‐delivery strategy would not affect the establishment of bacterial infection. Second, this co‐delivery strategy could make nanoparticles and bacteria to be better co‐dispersed at the sites of infection, achieving a stable and reliable therapeutic effect upon US stimulation. Third, mice need to be anesthetized to achieve lung delivery, and this co‐delivery strategy will reduce the number of times of anesthetization, meeting the 3R principle of animal research. All animal experiment protocols were reviewed and approved by the Institutional Animal Care and Use Committee of the Academy of Military Medical Sciences (Permit number: IACUC‐DWZX‐2023‐P025).

A sublethal pneumonia model was established by challenging mice with 2 × 10^5^ colony‐forming units (CFUs), which was the predetermined maximum *P. aeruginosa* dose leading to no death (LD_0_). US stimulation was applied on 24 h post bacterial inoculation to launch anti‐infective therapy. First, bacterial loads in the lung, liver, and spleen were determined by plate counting method once infective therapy was applied (Figure 5c,d). Abundant bacterial loads were detected in the lungs for the three control groups Mock, NP^FCPS^, and NP^FCPS‐P^, indicating the successful establishment of lung infections. By contrast, the NP^FCPS‐P^+US, NP^FCPS^+US, and US groups gave the statistically different inhibition rates at 99.3%, 86.8%, and 48.2%, respectively, indicating their very quick bactericidal actions. Meanwhile, a very small number of bacteria was detected in the liver and spleen, indicating that only a small portion of bacteria could enter into the bloodstream from the lung; there was statistically significant difference in inhibition rates for all those 6 groups, which might be due to the presence of quite too few bacteria (Figure S29, Supporting Information). Second, H&E staining was performed to observe the histopathologic changes in the 5 major organs (lung, heart, liver, spleen, and kidney) on days 0, 2, and 4 post‐treatment. On day 0, high levels of neutrophil infiltration and local hemorrhage with a pathological score of Grade 4 were found in the lung for all the 6 groups; on day 2 or 4, the NP^FCPS‐P^+US group gave the lowest levels of inflammatory response and pathological score in the lungs (Figure 5e,f). On days 0, 2, and 4 post‐therapy, H&E staining of the other four organs showed no obvious pathologic lesions (Figure S30, Supporting Information), confirming that the infection was restricted in the lung and few bacteria could spill into the bloodstream but cleared by mouse immune system.

An additional lethal pneumonia model was established by challenging mice with 7 × 10^5^ CFUs, which was the predetermined *P. aeruginosa* absolute lethal dose (LD_100_). US‐triggered anti‐infective therapy was launched on 2 h post bacterial inoculation. All mice died on day 3 post‐therapy for the three control groups Mock, NP^FCPS^, and NP^FCPS‐P^. By contrast, the NP^FCPS‐P^+US, NP^FCPS^+US, and US groups gave statistically different survival rates at 90%, 50%, and 20%, respectively (Figure 5g); meanwhile, the body weight curves gave no statistically significant difference between these 3 groups (Figure 5h).

Taken together, NP^FCPS‐P^+U had the best and most highly effective anti‐infective action against *P. aeruginosa*‐induced sublethal or lethal pneumonia in mice, and the therapeutic efficacies gave the trend as expected: NP^FCPS‐P^+US > NP^FCPS^+US > US.

### 2.6 Mechanisms of Anti‐Infective Action Viewed from Host Response

**Figure 6:**
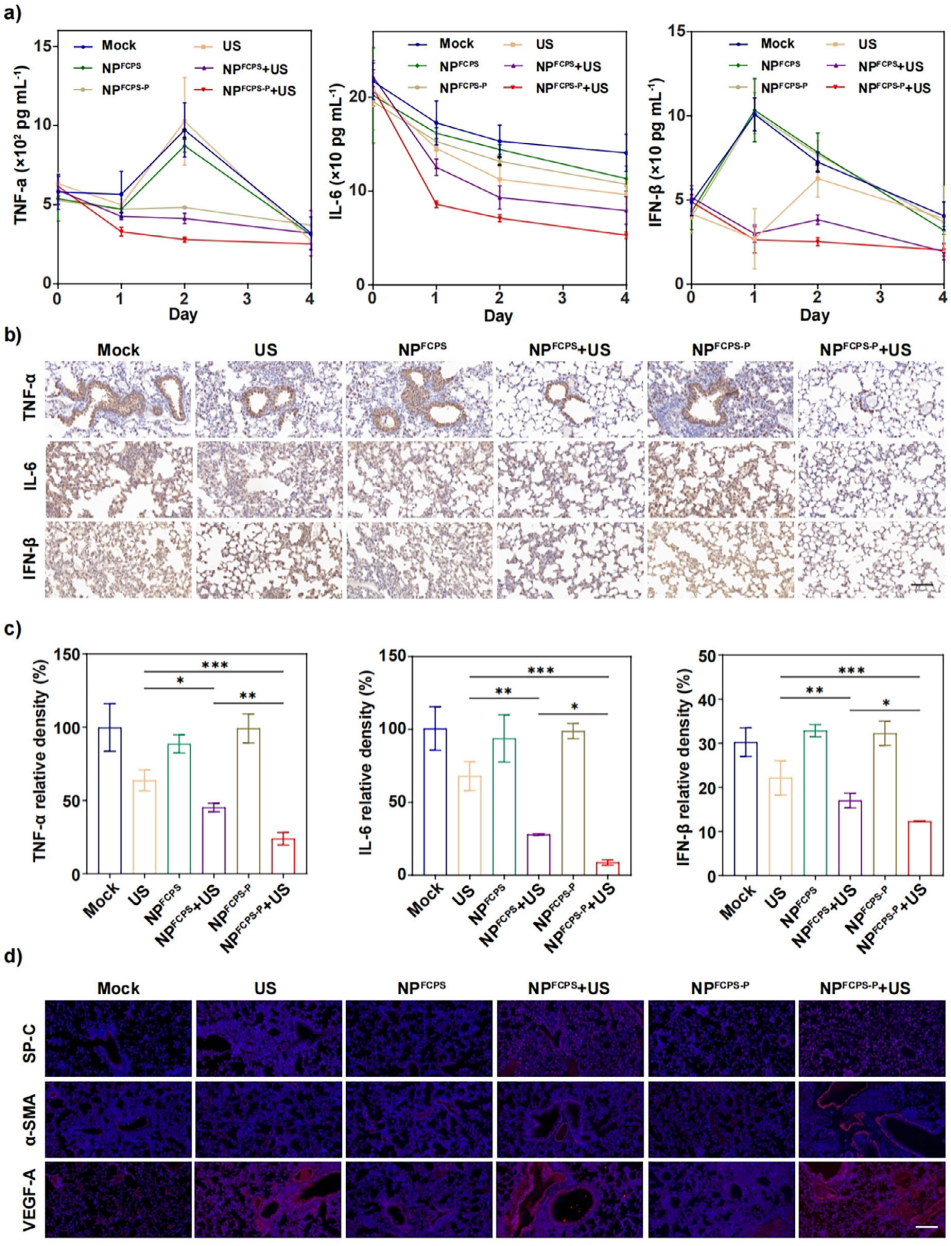
ELISA, IHS, and IFS assays of proinflammatory cytokines and lung injury repair proteins. US stimulation: 1.0 MHz, 1.5 W cm^−2^, 50% duty cycle, 2 min. a) Concentrations of proinflammatory cytokines TNF‐α, IL‐6, and IFN‐β in bronchoalveolar lavage fluids on days 0, 1, 2, and 4 post‐therapy. b) Representative IHS photographs and c) corresponding statistical analyses for expression levels of TNF‐α, IL‐6, and IFN‐β in the lungs on day 2 post‐therapy. Yellow‐brown represents positive detection. Scale bar = 100 µm. d) Representative IFS photographs and e) corresponding statistical analyses for expression levels of lung injury repair proteins SP‐C, α‐SMA, and VEGF‐A on day 2 post‐therapy. Red represents positive detection. Data are expressed as mean ± SD (n = 3). ns: *P* ≥ 0.05, ^*^: *P* < 0.05, ^**^: *P* < 0.01, ^***^: *P* < 0.001, one‐way ANOVA with Tukey's multiple comparisons test.

Mechanisms of in vivo anti‐infective action were systematically explored from the view of immunological and transcriptomic response in the lungs post‐therapy of sublethal *P. aeruginosa*‐induced infection. First, to characterize the expression of three major proinflammatory cytokines TNF‐α, IL‐6, and IFN‐β, the bronchoalveolar lavage fluids on days 0, 1, 2, and 4 post‐therapy were collected for enzyme‐linked immunosorbent assay (ELISA) (**Figure** **6**a). With the days went on, the expression of these cytokines was progressively decreased in the NP^FCPS‐P^+US group. As for the comparison of different treatment groups, day 2 post‐therapy represented the ideal time point, at which the NP^FCPS‐P^+US group gave the lowest expression of each cytokine and moreover the expression levels of all these cytokines displayed the statistically different trend as expected: NP^FCPS‐P^+US < NP^FCPS^+US < US. Second, the lung tissues on day 2 post‐therapy were subjected to immunohistochemical staining (IHS) for detecting these 3 cytokines (Figure 6b,c), confirming the ELISA results. Third, the lung tissues on day 2 post‐therapy were also used for immunofluorescence staining (IFS) to determine the 3 major lung injury repair proteins, namely SP‐C (generation of alveolar type II epithelial cells^[^ ^27^ ^]^), α‐SMA (generation of myofibroblasts and deposition of extracellular matrix^[^ ^28^ ^]^), and VEGF‐A (angiogenesis^[^ ^29^ ^]^) (Figure 6d and Figure S31, Supporting Information). The NP^FCPS‐P^+US group gave the highest expression of each protein, with the statistically different trend for the three treatment groups: NP^FCPS‐P^+US > NP^FCPS^+US > US. In conclusion, NP^FCPS‐P^+US had the best and highly effective action to inhibit the inflammatory response and meanwhile promote lung injury repair.

**Figure 7:**
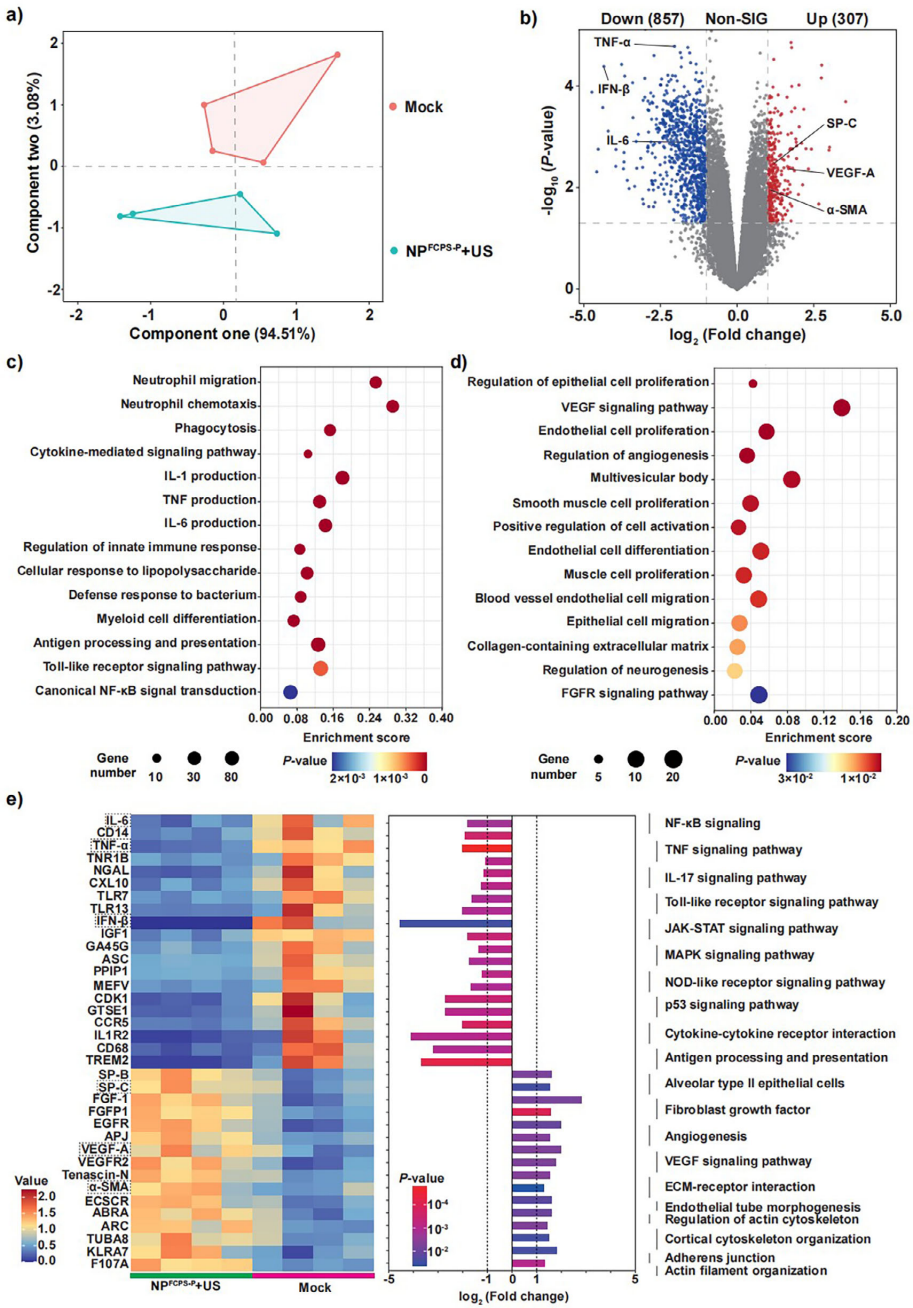
RNA‐seq assay of infected lung tissues on day 2 post‐therapy. a) PCA plot of clustered datasets between the two treatment groups NP^FCPS‐P^+US and Mock. US stimulation: 1.0 MHz, 1.5 W cm^−2^, 50% duty cycle, 2 min. b) Volcano plot of DGEs for NP^FCPS‐P^+US compared to Mock. Up: Up‐regulated genes. Down: Down‐regulated genes. Non‐SIG: non‐significantly differentially regulated genes. GO pathway enrichment analysis was performed for c) Down‐regulated and d) up‐regulated genes. e) Heat map of selected DRGs involved in inhibited inflammatory response and accelerated lung injury repair. Boxes indicate the 6 DRGs revealed simultaneously by IHS and RNA‐seq. Experiments were performed with 4 independent biological replicates.

In addition, the RNA‐seq transcriptomes of infected lung tissues on day 2 post‐therapy were compared between the two treatment groups NP^FCPS‐P^+US and Mock (**Figure** **7**). First, the PCA plot (Figure 7a) showed a high level of data reproducibility within each group and a high degree of data difference between the two groups. Second, a total of 857 down‐regulated genes (including the above 3 proinflammatory cytokines) and 307 up‐regulated genes (including the above 3 lung injury repair proteins) were identified for NP^FCPS‐P^+US in comparison to Mock (Figure 7b). Third, Gene Ontology (GO) pathway enrichment analysis of these DEGs identified not only the 14 down‐regulated pathways (Figure 7c; neutrophil migration, neutrophil chemotaxis, phagocytosis, cytokine‐mediated signaling pathway, IL‐1 production, TNF production, IL‐6 production, regulation of innate immune response, cellular response to lipopolysaccharide, defense response to bacterium, myeloid cell differentiation, antigen processing and presentation, Toll‐like receptor signaling pathway, and canonical NF‐κB signal transduction), but also the 14 up‐regulated pathways (Figure 7d; regulation of epithelial cell proliferation, VEGF signaling pathway, endothelial cell proliferation, regulation of angiogenesis, multivesicular body, smooth muscle cell proliferation, positive regulation of cell activation, endothelial cell differentiation, muscle cell proliferation, blood vessel endothelial cell migration, epithelial cell migration, collagen‐containing extracellular matrix, regulation of neurogenesis, and FGFR signaling pathway). Notably, all these enriched down‐regulated pathways contributed to the resolution of hyperinflammation, whereas all these enriched up‐regulated pathways were responsible for the promotion of lung injury repair, providing a global perspective on clearance of bacterial infection along with restoration of damaged lung. Fourth, as displayed by the heat map (Figure 7e), an array of 20 key DEGs were arbitrarily selected from the above‐enriched pathways, and they would represent the principal host‐responsive contributors to endow the treatment outcome of NP^FCPS‐P^+US. In conclusion, as viewed from the host immunological and transcriptomic response, the excellent therapeutic effect of NP^FCPS‐P^+US depended largely on the suppression of inflammatory response and simultaneously the acceleration of lung injury repair.

## 3 Conclusion

In summary, we developed an inhalable polymeric nanoparticle NP^FCPS‐P^ for the treatment of acute bacterial pneumonia. P^FCPS‐P^ was a pseudo‐conjugated polymer contained FCPS‐P (manifested as P^+^Ph_3_Br^−^‐modified sonosensitizer FCPS) as well as ROS‐sensitive thioketal bonds, and it was wrapped by DSPE‐mPEG_2000_ in an aggregated fashion to locate at a central position of NP^FCPS‐P^. NP^FCPS‐P^ displayed an excellent sonodynamic effect, generating ^1^O_2_ upon US stimulation in a time‐dependent manner. The generated ^1^O_2_ triggered the degradation of NP^FCPS‐P^ and the release of FCPS‐P. The released cationic FCPS‐P targeted to bacterial surface through electrostatic interaction and achieved bacterial killing under a synergistic action of SDT and P^+^Ph_3_Br^−^. NP^FCPS‐P^+US gave >90% in vitro inhibition rates against MDR ESKAPE pathogens, and it could cause extensive morphological and biochemical changes in bacteria, especially including morphological shrinkage and concavity, leakage of cytoplasmic contents, elevated DNA damage, and decreased cellular GSH levels. A metabolomics assay of *P. aeruginosa* revealed that NP^FCPS‐P^+US could lead to a comprehensive disorder in bacterial metabolism, particularly including suppressed biofilm formation and virulence, excessive ROS production and enhanced oxidative stress, inhibited glycolysis and TCA cycle, and inhibited DNA, mRNA, and tRNA biosynthesis. The ROS‐sensitive property of NP^FCPS‐P^ endowed its highly favored biosafety at cellular and animal levels. When NP^FCPS‐P^ was delivered in the lungs of mice, US stimulation could promote the diffusion of delivered NP^FCPS‐P^ and its SDT‐based ROS generation ability in the lungs. In mice, NP^FCPS‐P^+US exhibited a 99.3% bactericidal rate in *P. aeruginosa*‐induced sublethal pneumonia and rendered a 90% animal survival rate in lethal pneumonia. As disclosed by immunological staining and transcriptomics analyses, the excellent therapeutic effect of NP^FCPS‐P^+US depended largely on the suppression of pro‐inflammatory response and simultaneously acceleration of lung injury repair in mice. NP^FCPS‐P^+US represented a promising antibiotics‐alternative strategy for treating deeply seated infections caused by MDR bacteria.

## Conflict of Interest

The authors declare no conflict of interest.

## Supporting information



Supporting Information

## Data Availability

The data that support the findings of this study are available from the corresponding author upon reasonable request.
